# Association between PFAS compounds in follicular fluid and POR and the intervention effect of Qizi Yusi Tang: a study based on targeted PFAS quantification analysis

**DOI:** 10.3389/fendo.2026.1758118

**Published:** 2026-02-27

**Authors:** Hengbing Li, Jiacheng Li, Wenjing Jiang, Xin Hu, Zizhen Guo, Jingyan Song, Zhengao Sun

**Affiliations:** 1 The First Clinical Medical College, Shandong University of Traditional Chinese Medicine, Jinan, China; 2 Department of Thoracic Surgery, Qilu Hospital of Shandong University, Jinan, China; 3 School of Medicine, Tongji University, Shanghai, China; 4 Reproductive Center of Integrated Medicine, Affiliated Hospital of Shandong University of Traditional Chinese Medicine, Jinan, China

**Keywords:** non-randomized prospective sequential comparative design, poor ovarian response, Qizi Yusi Tang, targeted per- and polyfluoroalkyl substances analysis, traditional Chinese medicine

## Abstract

**Background:**

This study employs high-throughput targeted PFAS quantification analysis to analyze per- and polyfluoroalkyl substances (PFAS) metabolites in follicular fluid from women with Kidney Deficiency-Liver Stagnation Pattern poor ovarian response (POR) and IVF controls with normal ovarian reserve. Furthermore, we examine PFAS metabolite alterations in POR patients before and after intervention with kidney-tonifying and liver-soothing herbal medicine to suggest that PFAS compounds, particularly PFBA, are significant metabolic markers associated with the Kidney Deficiency-Liver Stagnation Pattern in POR.

**Methods:**

This study enrolled 93 patients undergoing *in vitro* fertilization and embryo transfer (IVF-ET). Participants were divided into three groups: (1) the non-herbal intervention group (POR-NON group, *n* =31); (2) the control group (POR-CON group, *n* =31); and (3) the herbal medicine intervention group (POR-TCM, *n* =31). And ultra-high performance liquid chromatography-triple quadrupole mass spectrometer (UHPLC-QTRAP MS) was used to detect metabolites in the three groups of samples.

**Results:**

Patients with Kidney Deficiency-Liver Stagnation Pattern POR showed significantly higher levels of PFBA in follicular fluid compared to IVF controls with normal ovarian reserve (P < 0.01). The level of PFBA in follicular fluid was significantly lower in patients with Kidney Deficiency-Liver Stagnation Pattern POR who received QZYST intervention (POR-TCM group) than in those who did not receive intervention (POR-NON group) (P < 0.05). QZYST intervention significantly reduced Kidney Deficiency-Liver Stagnation Pattern scores in POR patients compared to both baseline (P < 0.001) and the POR-NON group (P < 0.05). The POR-TCM group demonstrated significantly improved number of oocytes retrieved (P < 0.05) and two-pronuclei (2PN) zygotes (P < 0.05), along with significantly fewer cycles with no transferable embryos (P < 0.05) compared to the POR-NON group.

**Conclusions:**

Patients with Kidney Deficiency-Liver Stagnation Pattern POR exhibited significantly elevated levels of PFAS compounds in follicular fluid compared to IVF controls with normal ovarian reserve. Compared with the control group, patients who received QZYST intervention showed improvements in TCM syndrome manifestations and key clinical outcomes, and significantly lower PFBA levels were observed in the follicular fluid of the TCM intervention group than in those without intervention.

## Background

In recent years, IVF-ET has undergone significant advancements, offering hope to countless infertility patients. However, this technology also faces considerable challenges. During COS, a subset of patients exhibits suboptimal response to exogenous gonadotropins (Gn), characterized by: Limited follicular development; Low peak serum estradiol(E_2_) levels; High Gn dosage requirements; Reduced oocyte yield; Elevated cycle cancellation rate. These factors collectively contribute to diminished clinical pregnancy rates, defining a condition termed POR.

Epidemiological studies report a POR incidence of 5.6%–35.1% (1), accounting for approximately 9–26% of IVF indications (2). The 2011 Bologna Criteria established the first evidence-based framework for POR diagnosis and therapeutic strategies (3). In 2016, the Patient-Oriented Strategies Encompassing Individualized Oocyte Number (POSEIDON) group introduced a novel stratification system, categorizing patients by age, ovarian reserve markers and previous stimulation response. This paradigm shift replaced the term “poor response” with “low prognosis” (POSEIDON Group 1–4), substantially reducing interpatient heterogeneity (4).

The etiology of POR remains unclear but may involve advanced maternal age, genetic predisposition, prior ovarian surgery, pelvic inflammatory disease, and environmental factors (5), with the latter emerging as a key research focus in recent years (6). Clarifying the pathogenesis of POR and developing targeted prevention and treatment strategies to obtain an optimal number of high-quality oocytes during COS, thereby improving IVF success rates in POR patients, represents a critical challenge in reproductive medicine.

Although traditional Chinese medicine (TCM) does not specifically mention the disease term “POR,” its clinical manifestations can generally be categorized under TCM syndromes such as “infertility,” “scanty menstruation,” “delayed menstruation,” “amenorrhea,” and “menopausal syndrome.” Clinically, POR patients most frequently present with a Kidney Deficiency-Liver Stagnation Pattern (7, 8). Professor Zhengao Sun, a leading TCM expert, proposes that POR pathogenesis is closely related to liver-kidney dysfunction, consistent with the TCM theory of “simultaneous liver-kidney disorders,” with Kidney Deficiency-Liver Stagnation Pattern being the core pathogenesis. The clinical application of QZYST, a kidney-tonifying and liver-regulating herbal formula that nourishes blood and activates blood circulation, has demonstrated efficacy in improving POR patients’ clinical symptoms, ovarian reserve function, and ovarian responsiveness.

PFAS, a class of widespread and persistent EEDs found in various consumer products, can accumulate in human tissues through multiple exposure routes, posing significant health risks including reproductive toxicity (9). Transport proteins such as serum albumin, solute carrier proteins, and ATP-binding cassette transporters facilitate PFAS distribution throughout the body, enabling their penetration through the follicular barrier and subsequent detection in female follicular fluid (10). Emerging evidence indicates these compounds disrupt steroidogenesis by altering the expression of key steroidogenic enzymes (11), thereby impairing follicular development, oocyte maturation, and ultimately leading to diminished ovarian reserve and compromised oocyte quality (12). While current evidence suggests a potential association between PFAS exposure and POR, this relationship remains to be conclusively established and warrants further investigation.

Follicular fluid, serving as the critical microenvironment for oocyte growth and development, exhibits metabolic profiles intrinsically linked to oocyte and granulosa cell quality (13). Consequently, investigating metabolite dynamics in follicular fluid holds significant diagnostic and therapeutic value for conditions like POR. The advent and rapid advancement of metabolomics technologies have enabled robust quantification of follicular fluid metabolites, providing insights into oocyte developmental potential (14) and offering transformative opportunities for elucidating POR pathogenesis and guiding clinical management. This study utilizes high-throughput targeted PFAS quantification analysis to identify differential PFAS metabolite profiles in follicular fluid between POR patients with Kidney Deficiency-Liver Stagnation Pattern and IVF controls with normal ovarian reserve, and analyze PFAS level alterations following intervention with kidney-tonifying and liver-regulating herbal medicine (QZYST), and investigate the correlation between follicular fluid PFAS compounds and the pathogenesis of Kidney Deficiency-Liver Stagnation Pattern POR, thereby advancing the mechanistic understanding of POR development.

## Materials and methods

### Materials

#### Reagents and experimental instruments

Methanol (CNW Technologies, 67-56-1); Acetonitrile (CNW Technologies, 75-05-8); Ammonium hydroxide (Fisher Scientific, 1336-21-6); Ammonium acetate (Sigma-Aldrich, 631-61-8); Heptafluorobutyric acid (PFBA) (J&K Scientific, 375-22-4); Perfluorooctanoic acid (PFOA) (J&K Scientific, 335-67-1); Perfluorononanoic acid (PFNA) (J&K Scientific, 375-95-1); Perfluorooctane sulfonate (PFOS) (J&K Scientific, 1763-23-1); Perfluoroundecanoic acid (PFUnDA) (J&K Scientific, 2058-94-8); Perfluorodecanoic acid (PDA) (J&K Scientific, 335-76-2); 6500 Q-trap mass spectrometer (SCIEX); ExionLC™ System for UHPLC (SCIEX); Fresco™ 17 Microcentrifuge(Thermo Fisher Scientific); BSA124S-CW Analytical Balance (Sartorius); Mingche™ D 24UV (Merck Millipore).

#### Preparation of medicine and QZYST

Recombinant follicle-stimulating hormone alpha (r-hFSH-alfa)(Gonal F, Merck Serono S.p.A, Modugno, Italy); Recombinant follicle-stimulating hormone beta (r-hFSH-beta)(PUREGON, Merck & Co., Inc., Beijing, China), Human menopausal gonadotropin (HMG, Livzon Pharmaceutical Group Inc., Guangdong, China), Cetrorelix acetate (GnRH-ant, Baxter Oncology GmbH, North Rhine-Westphalia, Germany); Human chorionic gonadotropin (hCG, Livzon Pharmaceutical Group Inc., Guangdong, China); Recombinant hCG (rhCG, Ovidrel, Merck Serono S.p.A., Modugno, Italy).

QZYST was provided by the Affiliated Hospital of Shandong University of Traditional Chinese Medicine (Jinan, China) and authenticated by Dr. Zhengao Sun (Shandong University of Traditional Chinese Medicine). Its composition is as follows: Shudihuang (*Rehmanniae Radix Praeparata*)15g; Lujiaoshuang (*Cornu Cervi Degelatinatum*)15g; Roucongrong (*Cistanches Herba*)12g; Mohanlian (*Ecliptae Herba*)12g; Yujin (*Curcumae Radix*)12g; Gouqizi (*Lycii Fructus*)9g; Nvzhenzi (*Fructus Ligustri Lucidi*)9g; Sangshen (*Fructus Mori*)9g; Fupenzi (*Rubi Fructus*)9g; Tusizi (*Cuscutae Semen*)9g; Lianzixin (*Nelumbinis Plumula*)9g; Jinyingzi (*Rosae Laevigatae Fructus*)9g; Zhigancao (*Radix Glycyrrhizae*)6g. The decoction was prepared by boiling the herbs in water (200 mL per dose) and administered orally twice daily (morning and evening), 30 minutes after meals, with one dose per day. The decoction was consumed warm.

#### Participants

Patients who underwent IVF-ET treatment at the Reproductive and Genetics Center of the Affiliated Hospital of Shandong University of Traditional Chinese Medicine from January 2019 to September 2021 and met the inclusion criteria were enrolled as the non-TCM intervention group (POR-NON group, *n* = 31). A cohort of women undergoing IVF-ET solely due to male-factor infertility, characterized by normal ovarian reserve markers, was enrolled as the control group (POR-CON group, n = 31). Additionally, patients receiving IVF-ET at the same center from October 2021 to October 2022 who fulfilled the inclusion criteria were assigned to the TCM intervention group (POR-TCM group, *n* = 31).

#### TCM symptom pattern identification standard

Participants with POR were diagnosed with Kidney Deficiency-Liver Stagnation Pattern according to nationally standardized criteria. The diagnosis required(a) A minimum of two primary symptoms from the following: scanty menstruation, darkish or dull menstrual blood; premenstrual irritability or chronic depression; soreness in lumbar and knees;(b) At least two secondary symptoms including hot flashes with sweating; mental fatigue and weakness, insomnia with dreamfulness; dizziness and tinnitus; hypochondriac distension/pain; premenstrual breast distension; loss of libido; clear profuse urine;(c) Characteristic signs reflected by a pale tongue with thin white coating and a deep, thready-wiry pulse.

#### Diagnostic criteria of infertility and POR

Infertility, defined as the inability to conceive after at least 12 months of consistent, unprotected sexual activity (15). Participants were diagnosed with POR strictly based on the Bologna Criteria established by ESHRE in 2011 (3). To ensure a homogenous study population, a diagnosis of POR required the presence of at least two of the following: (a) Advanced maternal age (≥40 years) or other risk factors for POR; (b) A previous history of POR (≤3 oocytes retrieved in a conventional stimulation cycle); (c) Impaired ovarian reserve markers, specifically an antral follicle count (AFC) < 5–7 or anti-Müllerian hormone (AMH) < 0.5–1.1 ng/mL.

#### Inclusion criteria

Participants were enrolled if they fulfilled all criteria: (a) Confirmed infertility and POR diagnosis; (b) TCM pattern of Kidney Deficiency-Liver Stagnation; (c) Age 21–40 years, partner’s semen parameters within normal limits (WHO 5th edition), and planned IVF-ET; (d) GnRH antagonist protocol for ovarian stimulation; (e) Body mass index (BMI) 18.5–25 kg/m²; (f) No hormonal therapy within 3 months pre-enrollment; (g) Signed informed consent with protocol compliance.

#### Exclusion criteria

Participants were excluded if they met any of the following: (a) Autoimmune/genetic causes of POR; (b) Congenital reproductive tract anomalies or known chromosomal abnormalities; (c) Comorbid gynecological/endocrine disorders, acute infections, psychiatric conditions, or severe systemic diseases (cardiovascular/hepatic/renal/hematopoietic); (d) Active viral infections (HIV/HBV/HCV/syphilis); (e) Hypersensitivity to trial drugs; (f) Documented poor adherence (< 80% medication compliance).

#### Adherence and discontinuation criteria

Medication adherence in the POR-TCM group was monitored by counting returned herbal pouches and through telephone follow-up. Adherence was defined as the consumption of at least 80% of the scheduled doses. Participants would be discontinued from the study if they: (a) experienced serious adverse events; (b) demonstrated poor adherence (< 80% of doses); (c) developed acute systemic illnesses; (d) requested to withdraw for personal reasons. In this study, all participants maintained over 80% adherence, and no serious adverse events were observed.

### Methods

#### Controlled ovarian hyperstimulation and QZYST intervention

All three patient groups underwent the GnRH antagonist protocol. Transvaginal ultrasonography was performed on menstrual cycle day 3 to assess bilateral AFC and size. Ovarian stimulation was initiated with recombinant follicle-stimulating hormone (rFSH, 150–300 IU/day) based on age, BMI, and baseline endocrine profiles. After 3–4 days of continuous stimulation, follicular development and serum sex hormone levels were re-evaluated. Cetrorelix acetate (Cetrotide^®^; 0.25 mg/day) was subcutaneously administered if dominant follicles reached ≥12 mm. Final oocyte maturation was triggered by subcutaneous injection of 250 μg recombinant human chorionic gonadotropin (r-hCG, Ovidrel^®^) when ≥1 follicle measured ≥18 mm with corresponding E^2^ levels. The POR-TCM group additionally received QZYST from day 5 of the preceding menstrual cycle until trigger day (total treatment: 31–33 days). Transvaginal oocyte retrieval under ultrasound guidance was performed 34–36 hours post-trigger in all groups. The choice of gonadotropin type (rFSH-α, rFSH-β, or hMG) was determined by the clinicians based on individual patient characteristics such as age, BMI, and ovarian reserve markers. There were no significant differences in the distribution of Gn types across the study groups, ensuring comparability.

#### Sample collection and extraction

Follicular fluid samples collected on oocyte retrieval day were centrifuged (3000 × g, 15 min, 4°C), with 1.5 mL supernatant aliquoted and cryopreserved at -80°C). For PFAS analysis, 150 μL samples were mixed with 450 μL ice-cold methanol (-40°C) containing isotope-labeled internal standards, followed by vortexing (1 min) and ultrasonication (20 min, ice bath). After incubation at -40°C for 2 h, extracts were centrifuged (12,000 × g, 15 min, 4°C). Then 400 μL supernatant was concentrated under nitrogen stream, reconstituted in 100 μL methanol: water (7:3, v/v), ultrasonicated (10 min), and centrifuged again (12,000 × g, 15 min, 4°C) prior to LC-MS/MS injection. Calibration standards were prepared by serial dilution of 10 mmol/L PFAS master mix in matrix-matched solution. Due to hemolysis interference, 5 samples were excluded, resulting in 88 valid samples: POR-NON (*n* =30), POR-CON (*n* =29), POR-TCM (*n* =29). All samples were transferred to Shanghai Biotree Biotech Co., Ltd. for targeted metabolomic analysis.

#### UHPLC-MS/MS conditions

Chromatographic separation was achieved using an ExionLC UHPLC system (SCIEX) equipped with a Waters ACQUITY UPLC BEH C18 column (100 × 2.1 mm, 1.7 μm). The mobile phase comprised: (A) 3 mmol/L ammonium acetate with 3 mmol/L ammonia in water, and (B) acetonitrile. Operating conditions included: column temperature 40°C, sample tray 4°C, and injection volume 5 μL. Mass spectrometric detection employed a SCIEX 6500 QTRAP + triple quadrupole system with IonDrive Turbo V ESI source in negative ionization mode under these parameters: Curtain Gas: 30 psi; IonSpray Voltage: -4500 V; Temperature: 400°C; Ion Source Gas 1: 45 psi; Ion Source Gas 2: 45 psi. Prior to sample analysis, MRM transitions were optimized via direct infusion of standard solutions. For each PFAS compound, the most intense precursor-product ion transition was selected for quantification, with secondary transitions used for confirmation. All data acquisition and quantitative processing utilized SCIEX Analyst Software (v1.6.3) and MultiQuant 3.03.

#### Observation indicators

Baseline characteristics—including age, BMI, infertility duration, gravidity, parity, number of abortions, serum AMH, basal estradiol (bE_2_), basal luteinizing hormone (bLH), basal follicle-stimulating hormone (bFSH), baseline antral follicle count (bAFC), and infertility type—were compared between: POR-NON vs. POR-CON groups, and POR-NON vs. POR-TCM groups.

Clinical evaluation indicators were compared between POR-NON and POR-TCM groups, including: (1) Ovarian stimulation parameters (Gn duration, Gn dosage); (2) hCG trigger-day profiles (serum E_2_, LH, progesterone(P) levels; follicles ≥14 mm; endometrial thickness); (3) Embryological outcomes (oocytes retrieved, 2PN zygotes and fertilization rate, cleaved 2PN embryos, 2PN cleavage rate, transferable embryo count and rate, high-quality embryo count/rate); (4) Cycle management (fresh embryo transfer cycles, cycles with no transferable embryos, frozen embryo transfer cycles); (5) Pregnancy outcomes (clinical pregnancy rate per fresh transfer, per frozen transfer, cumulative clinical pregnancy rate per oocyte initiated cycle).

During days 2–4 of the menstrual cycle preceding ovarian stimulation, both POR-NON and POR-TCM groups completed the Kidney Deficiency-Liver Stagnation Pattern score scale—developed according to the Guiding Principles for Clinical Research of New Chinese Medicines (Trial). From day 5 of the preceding cycle, the POR-TCM group received daily QZYST until the day of the hCG trigger, while the POR-NON group received no herbal intervention. Both groups underwent conventional GnRH antagonist protocols. On the hCG trigger day, syndrome scores were re-evaluated alongside serum E_2_, P, and LH measurements. Embryo transfer (fresh or frozen-thawed) was individualized based on clinical parameters. Upon pregnancy confirmation, participants underwent scheduled follow-ups until delivery. Any adverse events during treatment mandated immediate discontinuation, medical intervention, and trial withdrawal.

### Statistical analysis

Raw mass spectrometry data underwent comprehensive preprocessing: Metabolite features were first filtered to retain only those with < 50% missing values in any single group or across all groups. Subsequently, missing values were imputed by multiplying the minimum detected value by a random number between 0.1 and 0.5. Following data curation, rigorous quality assessment was performed using quality control (QC) samples (relative standard deviation, RSD < 30%) and principal component analysis (PCA) of technical replicates. Subsequently, multivariate statistical analyses were conducted. Finally, comprehensive bioinformatic visualizations were generated.

Statistical analyses were performed using SPSS 26.0 (IBM Corp). Normality of data distribution was assessed by the Shapiro-Wilk test. Normally distributed continuous variables across the three groups were compared using one-way ANOVA followed by Tukey’s post-hoc test. Non-normally distributed continuous variables (such as PFAS concentrations) were analyzed using the Kruskal-Wallis H test, with Dunn’s test (Bonferroni-adjusted) for pairwise comparisons. Categorical data were evaluated by Pearson’s χ^2^ test with adjusted Z-tests for post-hoc analysis. A two-tailed p-value < 0.05 was considered statistically significant. To assess the reliability of our findings given the sample size, a post-hoc power analysis was performed using G*Power 3.1.9.7. The achieved power (1-β) for the PFBA was 0.89, and for the clinical outcome of oocytes retrieved, it was 0.53.

## Results

### Baseline characteristics

This study included 93 patients allocated into three groups: POR-NON group (*n* = 31): non-TCM intervention cohort; POR-CON group (*n* = 31): blank control cohort; POR-TCM group (*n* = 31): TCM treatment cohort. As shown in **Table 1**, there was no significant difference in age, BMI, infertility duration, gravidity, parity, abortion, infertility type, and basic luteinizing hormone, estrogen levels between the POR-NON group and POR-CON group. (*P* > 0.05). The POR-NON group demonstrated significantly lower serum AMH levels compared to the POR-CON group (0.6 ± 0.28 vs 3.85 ± 0.91 ng/mL, *P* < 0.05). Compared to the POR-CON group (median 7.3 IU/L, IQR 6.53-8.98), the POR-NON group demonstrated significantly higher bFSH level (median 9.36 IU/L, IQR 7.5-11.3). And bAFC were significantly lower in POR-NON group (median 7, IQR 5-10) compared to POR-CON group (median 26, IQR 20-32; P < 0.05).

**Table 1:** Baseline characteristics of participants.

Baseline characteristics	POR-NON (*n* = 31)	POR-CON (*n* = 31)	POR-TCM (*n* = 31)
Age (years)	36 (33, 38)	35 (31, 37)	37 (33, 39)
BMI (kg/cm^2^)	22.55 ± 2.12	22.93 ± 1.73	22.69 ± 2.11
Infertility duration (years)	3(2, 7)	3(2, 5)	4 (2,6)
Gravidity (n)	1.13 ± 1.2	0.74 ± 0.89	1.03 ± 1.22
Parity (n)	0.52 ± 0.77	0.32 ± 0.48	0.39 ± 0.62
Abortion (n)	0.61 ± 0.8	0.42 ± 0.89	0.65 ± 0.95
Infertility type (n)			
Primary infertility	13/31 (41.94%)	14/31 (45.16%)	14/31 (45.16%)
Secondary infertility	18/31 (58.06%)	17/31 (54.84%)	17/31 (54.84%)
AMH (ng/mL)	0.6 ± 0.28	3.85 ± 0.91 ^*^	0.78 ± 0.44
bE_2_ (pg/mL)	41.2 (29.41, 58)	37.49 (30.38, 43.59)	36 (23.18, 43)
bLH (IU/L)	4.53 ± 2.23	5.43 ± 2.86	4.13 ± 2.08
bFSH (IU/L)	9.36 (7.5, 11.3)	7.3 (6.53, 8.98) ^*^	8.95 (7.14, 11.62)
bAFC (n)	7 (5, 10)	26 (20, 32) ^*^	8 (6, 10)

POR-NON group: non-TCM treatment; POR-CON group: with male factor alone; POR-TCM group: with TCM treatment; BMI, body mass index; AMH, Anti-Müllerian hormone; bE_2_, basal estradiol; bLH, basal luteinizing hormone; bFSH, basal follicle-stimulating hormone; bAFC, basal antral follicle count. Data were expressed as mean ± standard deviation or %. *P*-values were determined by One-way ANOVA or Kruskal-Wallis H test followed by appropriate post-hoc pairwise comparisons. Compared with the POR-NON group, ^*^P < 0.05.

### Clinical evaluation indicators

As shown in **Table 2**, there was no significant difference in Gn days, Gn dosage, E_2_ of hCG day, LH of hCG day, P of hCG day, 2PN fertilization rate, cleaved 2PN embryos, 2PN cleavage rate, rate of embryos available for transfer, high-quality embryos, high-quality embryo rate, fresh embryo transfer cycles, clinical pregnancy rate per fresh transfer embryo, frozen embryo transfer cycles, clinical pregnancy rate per frozen transfer embryo, and cumulative clinical pregnancy rate per initiated oocyte retrieval cycle between POR-TCM group and POR-NON group (*P* > 0.05). Compared to the POR-NON group, the POR-TCM group demonstrated significantly higher numbers of oocytes retrieved and 2PN zygotes, along with a significantly lower proportion of cycles with no transferable embryos (all *P* < 0.05).

**Table 2:** Clinical evaluation indicators of participants.

Clinical evaluation indicators	POR-NON (*n* = 31)	POR-TCM (*n* = 31)	*P-value*
Gn days (n)	9.77 ± 1.73	9 ± 1.55	0.068
Gn dosage (IU)	2750 (2250, 3225)	2700 (2100, 3000)	0.206
Gn types r-hFSH-alfa r-hFSH-beta HMG	12/31 (38.71%) 10/31 (32.26%) 9/31 (29.03%)	13/31 (41.94%) 10/31 (32.26%) 8/31(25.81%)	0.952
E_2_ of hCG day (pg/mL)	842.17 (557.49, 1399.77)	1200.29 (797.77, 1452.38)	0.068
LH of hCG day (IU/L)	1.77 ± 0.98	1.89 ± 1.07	0.653
P of hCG day (nmol/L)	0.86 ± 0.42	1.03 ± 1.02	0.395
Follicles ≥14 mm on hCG day (n)	3.68 ± 1.89	4.42 ± 1.75	0.113
Endometrial thickness of hCG day (mm)	10.79 ± 2.61	11 ± 2.29	0.746
Oocytes retrieved (n)	3.29 ± 1.27	4 ± 1.44	0.044
2PN zygotes (n)	2.65 ± 0.91	3.26 ± 1.29	0.035
2PN fertilization rate	82/102 (80.39%)	101/124 (81.45%)	0.858
Cleaved 2PN embryos (n)	2.03 ± 1.05	2.52 ± 1	0.067
2PN cleavage rate	63/82 (76.83%)	78/101 (77.23%)	1.000
Embryos available for transfer (n)	1.48 ± 0.85	1.9 ± 0.91	0.065
Rate of embryos available for transfer	46/63 (73.02%)	59/78 (75.64%)	0.626
High-quality embryos (n)	0.45 ± 0.57	0.61 ± 0.72	0.330
High-quality embryo rate	14/63 (22.22%)	19/78 (24.36%)	0.737
Fresh embryo transfer cycles	16/31 (51.61%)	18/31 (58.06%)	0.394
Cycles with no transferable embryos	5/31 (16.13%)	2/31 (6.45%)	0.024
Clinical pregnancy rate per fresh transfer embryo	7/16 (43.75%)	8/18 (44.44%)	1.000
Frozen embryo transfer cycles	10/31 (32.26%)	11/31 (35.48%)	0.653
Clinical pregnancy rate per frozen transfer embryo	3/10 (30%)	3/9 (33.33%)	0.648
Cumulative clinical pregnancy rate per initiated oocyte retrieval cycle	10/31 (32.26%)	11/31 (35.48%)	0.653

POR-NON group: non-TCM treatment; POR-CON group: with male factor alone; POR-TCM group: with TCM treatment; Gn, gonadotropin; hCG, human chorionic gonadotropin; E_2_, estradiol; LH, luteinizing hormone; P, progesterone; 2PN, two-pronuclei. 2PN fertilization rate = Number of 2PN zygotes/Number of oocytes retrieved 100%; 2PN cleavage rate = Number of cleaved 2PN embryos/Number of 2PN zygotes × 100%; Rate of embryos available for transfer = Number of embryos available for transfer/Number of cleaved 2PN embryos × 100%; High-quality embryo rate = Number of high-quality embryos/Number of cleaved 2PN embryos × 100%; Clinical pregnancy rate per fresh transfer embryo = Number of clinical pregnancies in fresh embryo transfer cycles/Number of fresh embryo transfer cycles × 100%; Clinical pregnancy rate per frozen transfer embryo = Number of clinical pregnancies in frozen embryo transfer cycles/Number of frozen embryo transfer cycles × 100%; Cumulative clinical pregnancy rate per initiated oocyte retrieval cycle = Number of clinical pregnancies from all related transfers/Number of initiated stimulation cycles × 100%. Data were expressed as mean ± standard deviation or %. *P*-values were determined by One-way ANOVA or Kruskal-Wallis H test followed by appropriate post-hoc pairwise comparisons.

### Kidney deficiency-liver stagnation pattern scores before and after treatment

The results demonstrated that both POR-TCM group and POR-NON group showed statistically significant improvements in Kidney Deficiency-Liver Stagnation Pattern scores after treatment (*P* < 0.01). However, the POR-TCM group receiving QZYST intervention achieved significantly greater Kidney Deficiency-Liver Stagnation Pattern score reduction compared to the non-TCM POR-NON group (*P* < 0.05). These findings indicate that QZYST effectively alleviates clinical symptoms in POR patients (**Table 3**).

**Table 3:** Comparison of kidney deficiency-liver stagnation pattern scores before and after treatment between POR-TCM group and POR-NON group.

Clinical evaluation indicators	POR-NON (*n* = 31)	POR-TCM (*n* = 31)	*P-value*
Total score before treatment	22 (20, 24)	21 (18, 24)	0.410
Total score after treatment	21 (19, 23)	18 (15, 22)	0.033
*P-value*	< 0.01	< 0.001	

POR-NON group: non-TCM treatment; POR-TCM group: with TCM treatment. Continuous data were represented as median (25th and 75th percentile) because of nonnormal distribution.

Compared to baseline, POR-TCM group demonstrated statistically significant improvements (*P* < 0.05) in: premenstrual irritability/chronic depression, soreness in lumbar and knees, dizziness and tinnitus, premenstrual breast distension, loss of libido, and dark tongue body. Although not statistically significant (*P* > 0.05), favorable clinical trends were observed in: scanty darkish menses, hot flashes with sweating, mental fatigue, insomnia with dreamfulness, hypochondriac distension, and clear profuse urine (**Table 4**).

**Table 4:** Changes in kidney deficiency-liver stagnation pattern scores in POR-TCM group before vs after treatment.

Syndrome	Score before treatment	Score after treatment	*P-value*
Scanty menstruation, darkish or dull menstrual blood	3.87 ± 1.71	3.55 ± 1.84	0.057
Premenstrual irritability or chronic depression	3.48 ± 1.55	2.97 ± 1.54	< 0.01
Soreness in lumbar and knees	3.55 ± 1.61	2.71 ± 1.83	< 0.01
Hot flashes with sweating	0.68 ± 0.87	0.58 ± 0.72	0.083
Mental fatigue and weakness	0.71 ± 0.78	0.61 ± 0.72	0.083
Insomnia with dreamfulness	0.48 ± 0.72	0.42 ± 0.67	0.161
Dizziness and tinnitus	0.81 ± 0.95	0.68 ± 0.79	0.043
Hypochondriac distension/pain	0.68 ± 0.83	0.61 ± 0.8	0.161
Premenstrual breast distension	1.06 ± 1	0.87 ± 0.85	0.031
Loss of libido	0.97 ± 0.95	0.71 ± 0.78	< 0.01
Clear profuse urine	1.45 ± 0.89	1.39 ± 0.88	0.161
Dark tongue body	0.71 ± 0.46	0.58 ± 0.5	0.043
Deep thready pulse	0.81 ± 0.4	0.74 ± 0.44	0.161
Wiry pulse	0.81 ± 0.4	0.74 ± 0.44	0.161

POR-TCM group: with TCM treatment. Data were expressed as mean ± standard deviation.

### Multivariate statistical analysis

#### Data quality assessment

**Figure 1:**
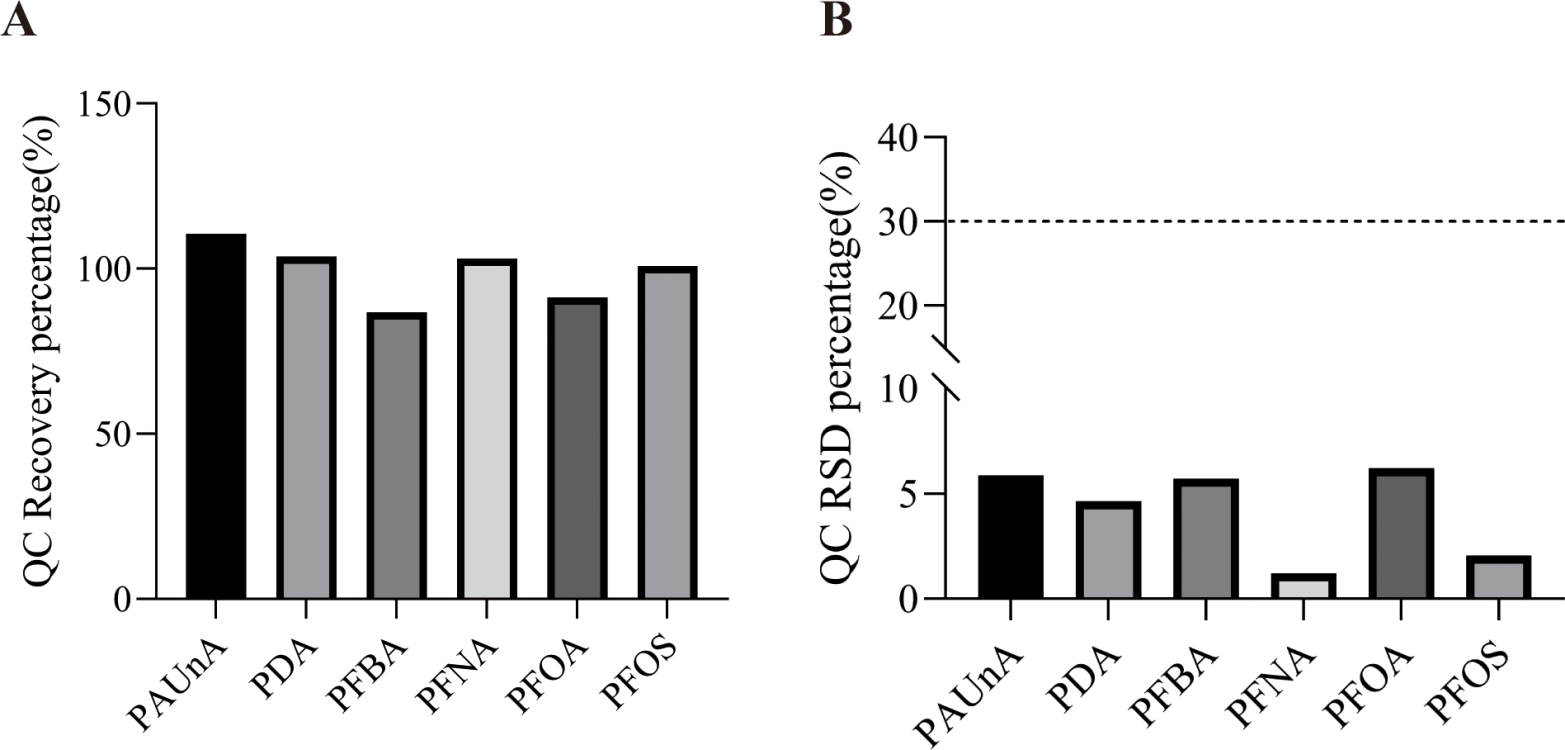
**(A)** Recovery rates and **(B)** RSD of QC samples for target PFAS compounds. QC, quality control; RSD, relative standard deviation.

Quantitative parameters for target metabolites are presented in **Table 5**. The lower limits of detection (LLODs) for target metabolites ranged from 0.01 to 0.10 nmol/L, while lower limits of quantification (LLOQs) ranged from 0.02 to 0.20 nmol/L. All target metabolites demonstrated correlation coefficients (R^2^) > 0.9979, indicating excellent linear relationships between chromatographic peak areas and metabolite concentrations, thereby meeting the requirements for targeted metabolomic analysis. Recovery rates and relative standard deviations (RSD) for quality control (QC) samples are detailed in **Table 6** and **Figure 1**. As shown in **Table 6**, the mean recoveries for all target metabolites were 86.82%-110.62% with RSD < 6.21%. These data confirm that the method accurately and reliably quantifies target metabolites within the specified concentration ranges, and the experimental results are suitable for subsequent research.

**Table 5:** Target metabolite quantification parameters.

Metabolite	LLOD(nmol/L)	LLOQ(nmol/L)	ULOQ(nmol/L)	R^2^
PAUnA	0.10	0.20	100.00	0.9979
PDA	0.05	0.10	50.00	0.9981
PFBA	0.10	0.20	200.00	0.9999
PFNA	0.10	0.20	50.00	0.9990
PFOA	0.05	0.10	100.00	0.9995
PFOS	0.10	0.20	100.00	0.9997

LLOD, lower limits of detection; LLOQ, lower limits of quantification; ULOQ, upper limit of quantification; R^2^, correlation coefficient.

**Table 6:** Sample recovery rate and RSD.

Metabolite	Concentration (nmol/L)	Recovery rate (%)	RSD (%)
PAUnA	20.0	110.62	5.87
PDA	20.0	103.65	4.64
PFBA	20.0	86.82	5.70
PFNA	20.0	103.11	1.22
PFOA	20.0	91.31	6.21
PFOS	20.0	100.81	2.05

RSD, relative standard deviation.

#### Comparison of follicular fluid metabolite relative quantitation values

As shown in **Table 7**, the results demonstrated that: PFBA levels in follicular fluid were significantly higher in the POR-NON group compared to POR-CON (mean: 1.28 vs 0.70 nmol/L; *P* < 0.01); Five metabolites (PDA, PFOS, PFNA, PFOA, PAUnA) showed upward trends in the POR-NON group, though without statistical significance (*P* > 0.05).Following QZYST intervention, the POR-TCM group exhibited significantly lower PFBA levels in follicular fluid compared to the POR-NON group (mean: 0.88 vs 1.28 nmol/L; *P* =0.016), while five other metabolites (PDA, PFOS, PFNA, PFOA, PAUnA) showed non-significant downward trends (all *P* > 0.05).

**Table 7:** Comparison of follicular fluid metabolite relative quantitation values.

Metabolite	POR-NON (nmol/L)	POR-CON (nmol/L)	POR-TCM (nmol/L)
PAUnA	1.56	1.50	1.24
PDA	1.07	0.83	0.78
PFBA	1.28	0.70^**^	0.88^*^
PFNA	1.29	1.21	1.23
PFOA	28.20	19.10	18.09
PFOS	5.62	4.82	4.58

Data were expressed as mean. *P*-values were determined by One-way ANOVA or Kruskal-Wallis H test followed by appropriate post-hoc pairwise comparisons. Compared with the POR-NON group, ^*^P < 0.05. Compared with the POR-NON group, ^**^P < 0.01.

### Principal component analysis

**Figure 2:**
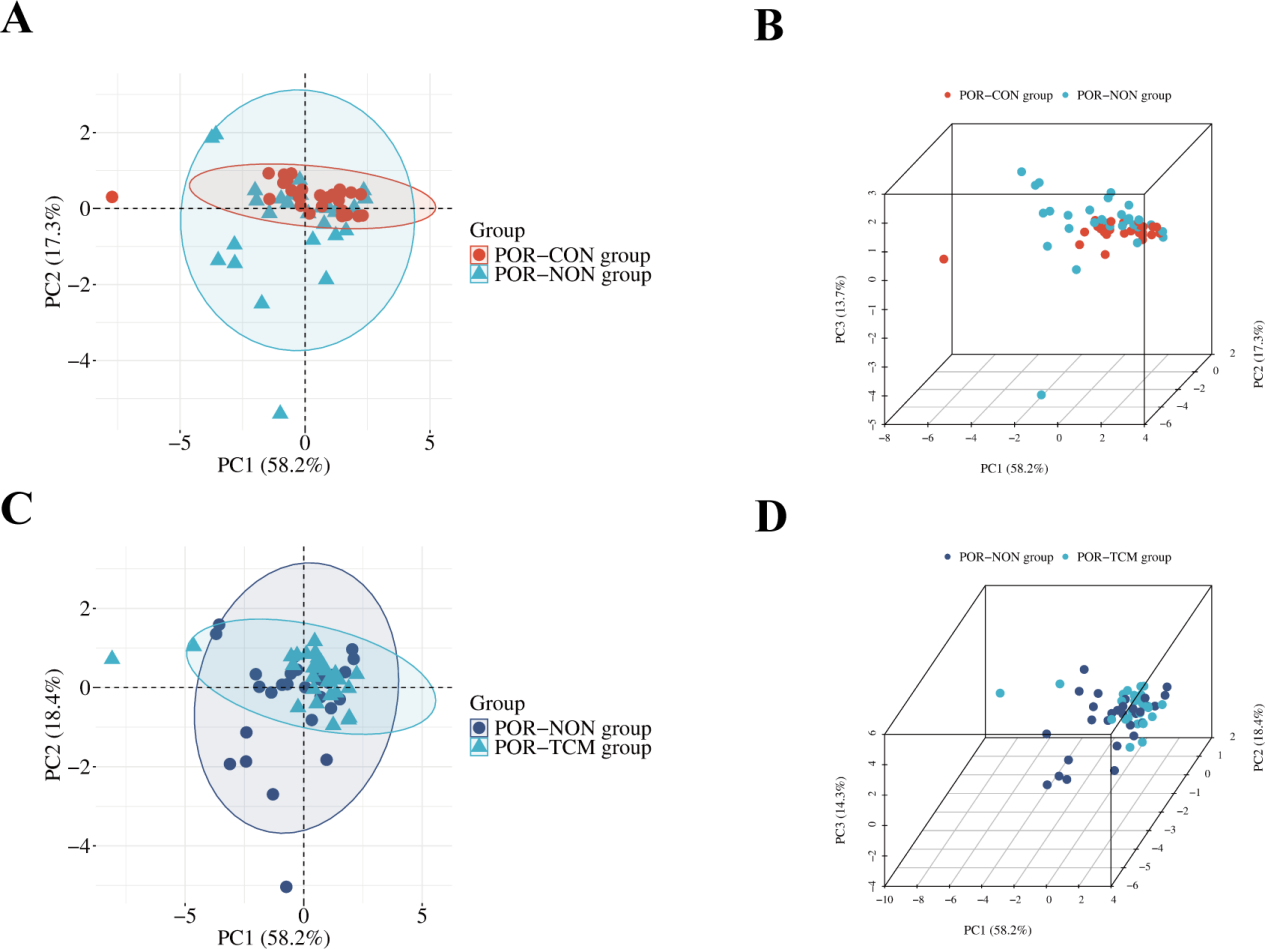
PCA plots of samples of each group. The x-axis (PC1), y-axis (PC2), and z-axis (PC3) represent the scores of the first, second, and third principal components, respectively. Each scatter point represents a sample; **(A, B)** PCA plots of the POR-CON group vs. the POR-NON group; **(C, D)** PCA plots of the POR-TCM group vs. the POR-NON group.

PCA revealed distinct clustering patterns (**Figure 2**), with both the POR-NON vs POR-CON and POR-NON vs POR-TCM comparisons demonstrating tight sample aggregation within their respective 95% confidence ellipses, indicating robust group separation.

### Orthogonal projections to latent structures- discriminant analysis

**Figure 3:**
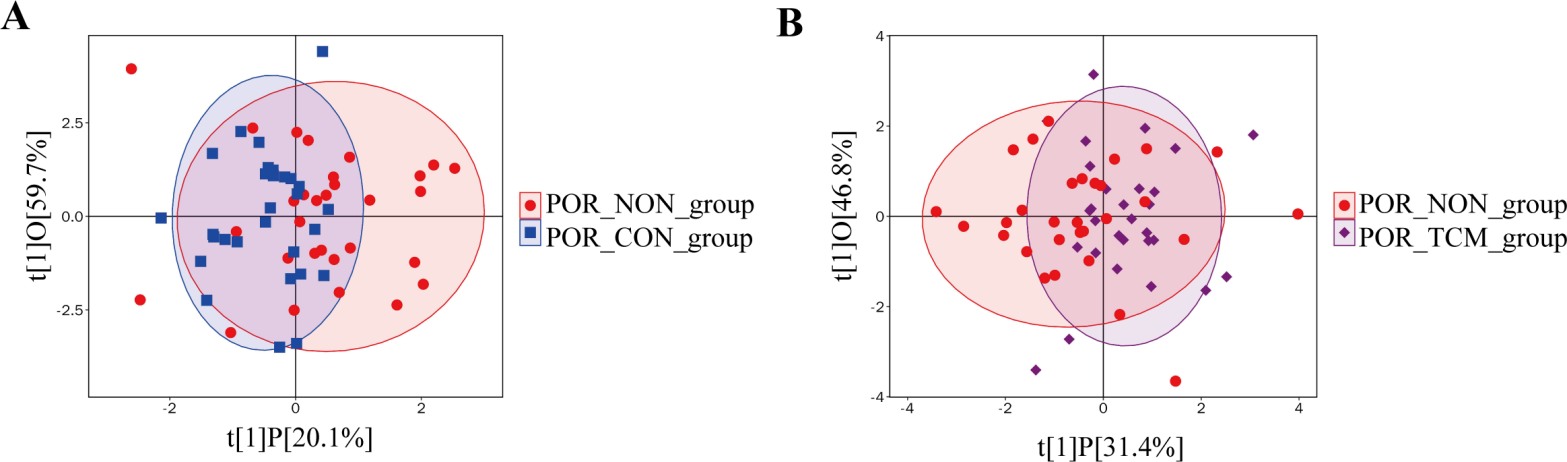
**(A)** OPLS-DA score plot showing discrimination between POR_NON and POR_CON groups. **(B)** OPLS-DA score plot showing discrimination between POR_NON and POR_TCM groups. The x-axis (t[1]P) represents predictive principal component scores for between-group variation, and the y-axis (t[1]O) represents orthogonal component scores for within-group variation. Each point corresponds to a sample, with shape and color indicating different experimental groups.

OPLS-DA analysis demonstrated partial but distinct separations between groups (**Figure 3**): the POR-NON group showed discernible separation from POR-CON, suggesting potential involvement of PFAS compounds in POR pathogenesis, while the separation between POR-TCM and POR-NON groups implied modulatory effects of QZYST intervention.

### Univariate analysis and correlation analysis

**Figure 4:**
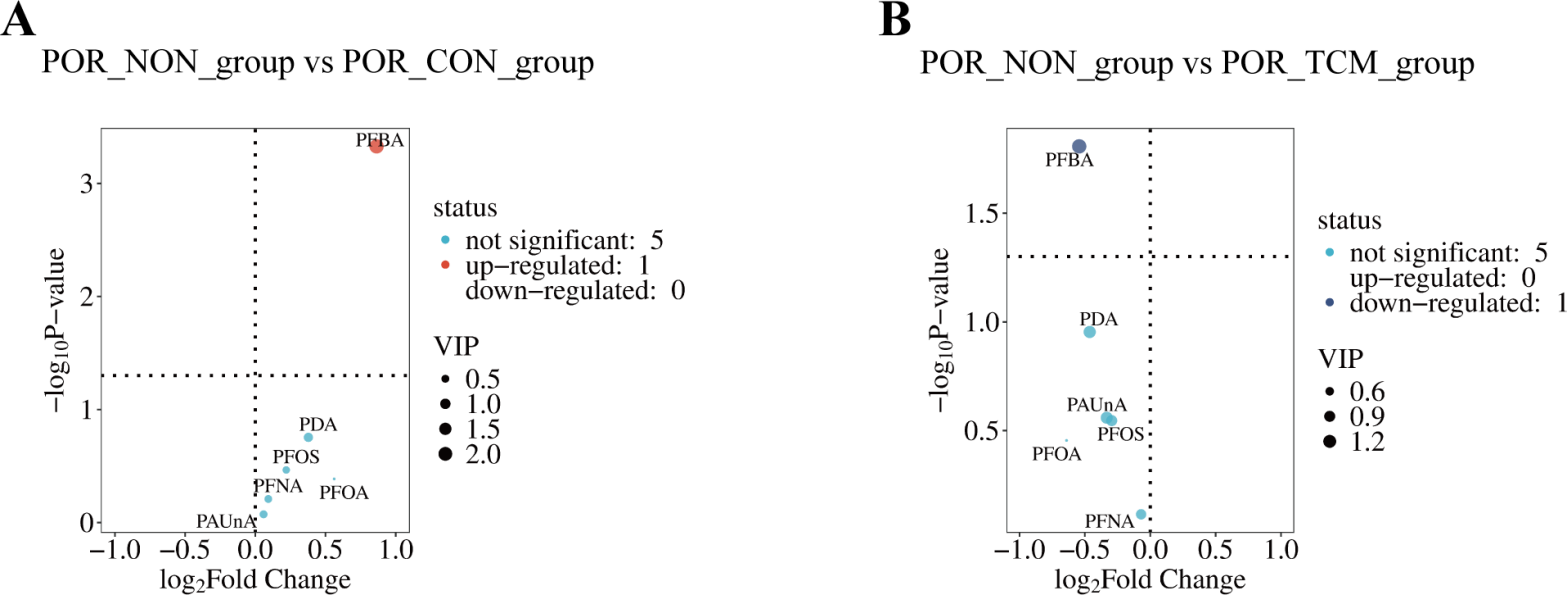
**(A)** Volcano plot of differential compounds between POR_NON and POR_CON groups. **(B)** Volcano plot of differential compounds between POR_NON and POR_TCM groups. Each point represents a compound. The x-axis shows log_2_ fold change between compared groups, while the y-axis displays -log_10_(*P-value*) from t-tests. Point sizes correspond to variable importance in projection (VIP) values from OPLS-DA modeling, with larger sizes indicating higher VIP scores.

**Figure 5:**
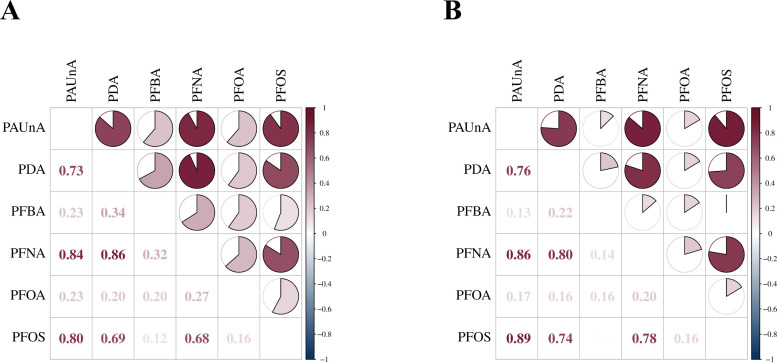
Correlation analysis heatmap. Correlation heatmap of the differential metabolites. **(A)** POR_CON vs. POR_NON. **(B)** POR_NON vs. POR_TCM.

Statistical analysis using UVA revealed distinct PFAS metabolite profiles in follicular fluid (**Figure 4**): Compared to POR-NON group, POR-TCM group showed elevated levels of all six PFAS compounds (PFBA, PDA, PFOS, PFNA, PFOA, PAUnA), with PFBA demonstrating statistically significant increase (*P* < 0.05). Following QZYST intervention, the POR-TCM group exhibited reduced PFAS concentrations relative to untreated POR-NON, with PFBA showing the most pronounced and statistically significant decrease (*P* < 0.05). The downward trends observed for the remaining five metabolites did not reach statistical significance (all *P* > 0.05). To elucidate interrelationships among the six PFAS, we conducted correlation analyses with visualization (**Figure 5**). Results demonstrated significant positive correlations across all study groups, suggesting synergistic interactions in POR pathogenesis.

### Hierarchical clustering analysis and relative concentration of metabolites

**Figure 6:**
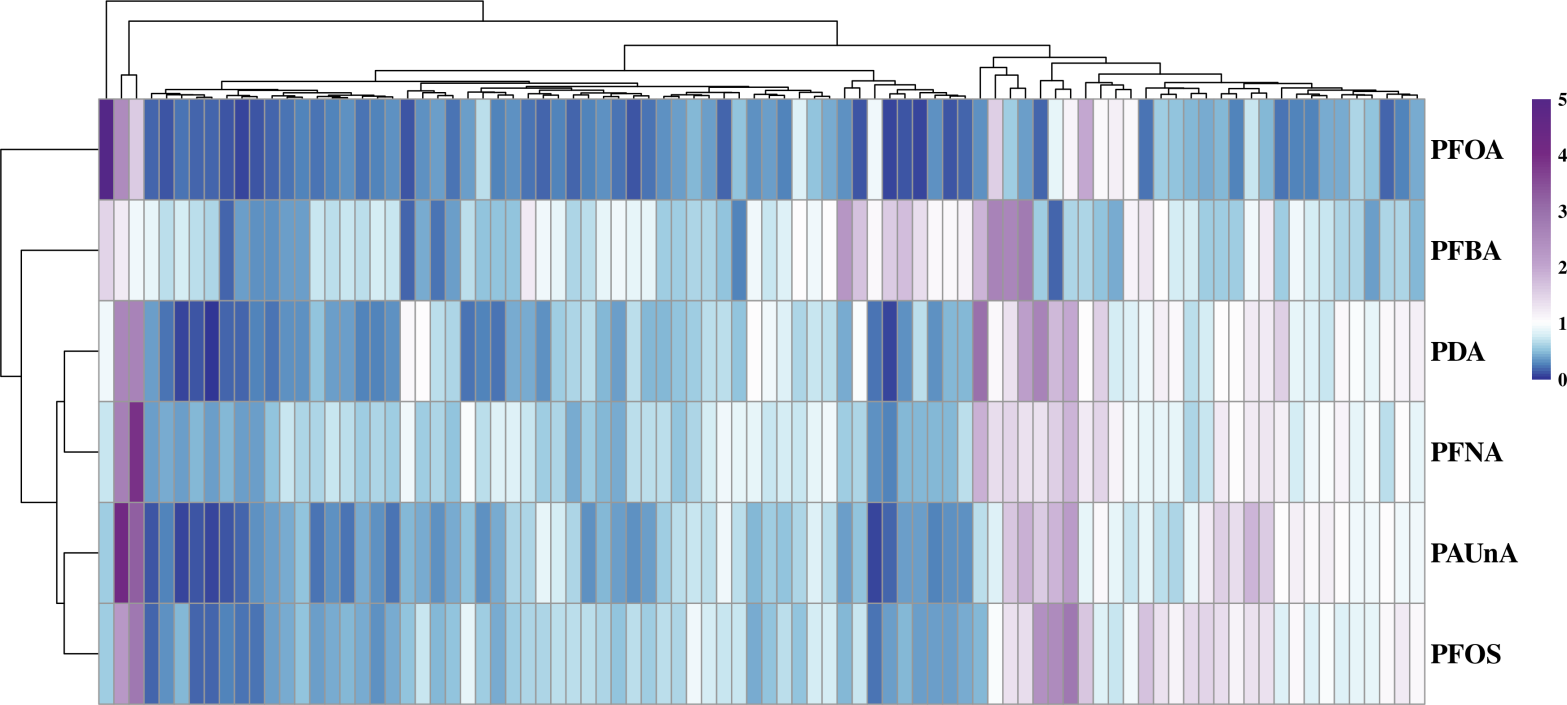
HCA heatmap plot of metabolites. The hierarchical clustering heatmap illustrates differentially abundant metabolites between experimental groups. The x-axis denotes the experimental groups, while the y-axis lists the identified metabolites. Color intensity within each tile represents the relative abundance of the corresponding metabolite: red indicates upregulation (higher abundance) and blue indicates downregulation (lower abundance) within the respective group.

**Figure 7:**
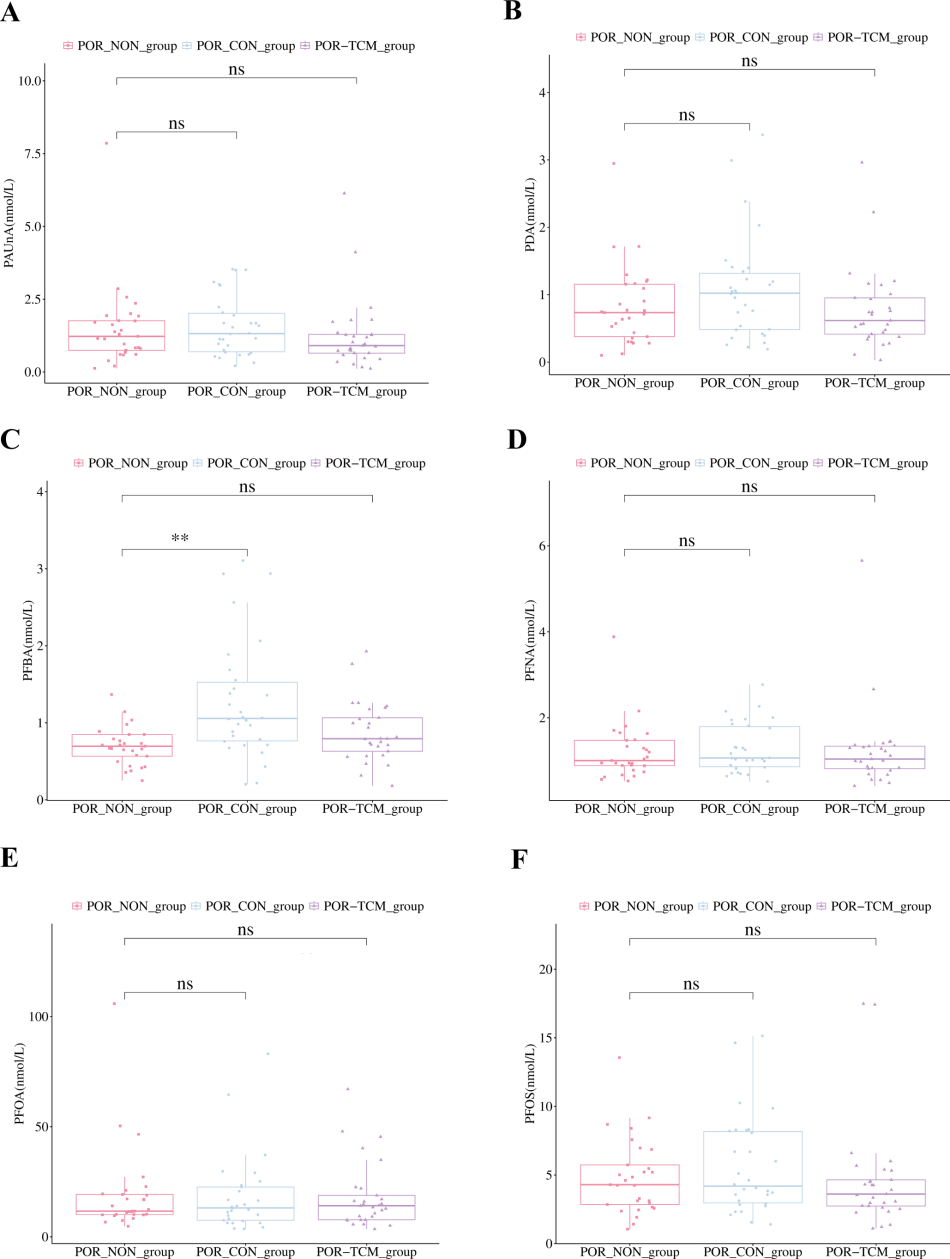
Boxplots showing the concentrations of PFAS compounds in the POR_NON, POR_CON, and POR_TCM groups. **(A)** PAUnA; **(B)** PDA; **(C)** PFBA; **(D)** PFNA; **(E)** PFOA; **(F)** PFOS. The x-axis represents the experimental groups, and the y-axis represents the concentration. Each point is colored and shaped according to its group. Compared with the POR_NON group: **P < 0.01; ns, *P* > 0.05 (not significant).

Based on the relative abundance data of follicular fluid metabolites across experimental groups, HCA was performed to generate a heatmap depicting the differentially abundant metabolites among groups (**Figure 6**). Additionally, box plot analysis provided a more intuitive visualization of intergroup differences in these metabolites (**Figure 7**).

## Discussion

This study enrolled 93 women undergoing IVF-ET, who were assigned in a non-randomized, prospective, sequential comparative manner to the POR-NON (n = 31), POR-CON (n = 31), and POR-TCM (n = 31) groups. Using high-throughput targeted PFAS quantification analysis, we quantified six PFAS in follicular fluid to investigate their role in POR pathogenesis. While prior studies have examined PFAS exposure in relation to blood to follicle transfer efficiencies (16), embryo quality (17), and fertility outcomes (18)—or employed untargeted metabolomics to explore associations between follicular fluid endocrine disruptors and POR (19–21)—this work provides preliminary evidence for an association between follicular fluid PFAS compounds and the development of POR, suggesting they may play a role in the pathological microenvironment of the follicle and demonstrate the clinical efficacy of TCM intervention for PFAS-exposed POR patients.

Follicular fluid—an ultrafiltrate of plasma reflecting systemic metabolic dynamics—constitutes the critical microenvironment for oocyte development, where its compositional profile directly influences oocyte and granulosa cell quality (22). PFAS, as pervasive EEDs in industrial and consumer products, demonstrably impair endocrine and reproductive functions (23–25). In this study, targeted quantification of six PFAS congeners revealed PFOA as the predominant compound across all groups (POR-CON, POR-NON, POR-TCM), followed by PFOS, aligning with prior epidemiological evidence (17).

PFBA, a short-chain PFAS with high water solubility and low bioaccumulation potential (elimination half-life: 48–96 hours (26)), is increasingly used as an alternative to long-chain compounds like PFOA/PFOS. However, its widespread detection in drinking water sources raises toxicological concerns (27, 28). Our study revealed significantly elevated PFBA levels in follicular fluid of Kidney Deficiency-Liver Stagnation Pattern POR patients versus controls (*P* < 0.01). Experimental evidence indicates PFBA induces hepatotoxicity across species (rodents, zebrafish, humans) by disrupting lipid metabolism and gene expression via peroxisome proliferator-activated receptor alpha (PPARα) activation. Bjork et al. demonstrated 100 μM PFBA upregulated Acox, Cte/Acot1, and Cyp4a1/11 in rat hepatocytes, yet lost PPARα-stimulatory effects at 200 μM in both rat and human liver cells (29). Foreman et al. confirmed PPARα-dependent hepatomegaly in humanized PPARα mice (30). Zebrafish studies showed sublethal PFBA exposure caused metabolic disruption and lipid droplet accumulation despite unchanged PPAR, Fabp3, and crot expression (31). We thus propose that PFBA contributes to POR pathogenesis by suppressing aromatase expression via PPARα signaling (32), thereby impairing oocyte maturation, developmental competence, and quality.

PDA, a long-chain PFAS with a 10-carbon backbone, exhibits heightened toxicity proportional to chain length (33). Consequently, PDA poses greater environmental and human health risks than shorter-chain congeners. Although follicular fluid PDA levels showed no statistically significant difference between Kidney Deficiency-Liver Stagnation Pattern POR patients and controls (*P* > 0.05), a marginally elevated trend was observed (1.07 vs. 0.83 nmol/L). Experimental evidence confirms PDA activates PPARα, upregulating Cyp2B10 and 4A14 expression in murine liver to induce hepatotoxicity (34). In primary mouse renal cells, PDA elevates intracellular reactive oxygen species (ROS) while suppressing superoxide dismutase activity, exacerbating oxidative stress and triggering apoptosis (35). Porcine oocyte studies further demonstrate PDA disrupts calcium homeostasis, impairing gap junctional communication between cumulus cells and oocytes, thereby compromising developmental competence and assisted reproductive technology (ART) success rates (36). Deng et al. revealed PDA inhibits maturation-promoting factors, causing meiotic arrest at prophase I and spindle assembly checkpoint dysfunction—accelerating meiotic progression and aneuploidy risk. Concurrent mitochondrial defects and ROS accumulation induce oocyte apoptosis (37). We thus propose PDA contributes to Kidney Deficiency-Liver Stagnation Pattern POR pathogenesis through PPARα-mediated aromatase suppression, oxidative stress induction, disruption of granulosa-oocyte gap junctions, and interference with meiotic fidelity.

PFOS, a prototypical and extensively studied PFAS compound, showed marginally elevated levels in the follicular fluid of Kidney Deficiency-Liver Stagnation Pattern POR patients compared to controls (5.62 vs. 4.82 nmol/L), though this difference lacked statistical significance (*P* > 0.05). Accumulating evidence indicates PFOS induces hepatorenal damage via oxidative stress (38, 39) and reduces fertilization potential, as evidenced by negative dose-response relationships in meta-analyses (40). Crucially, PFOS impairs oocyte competence through three synergistic mechanisms: (1) Disrupting cytoskeletal assembly to cause spindle aberrations, chromosomal misalignment, and actin dysfunction; (2) Compromising mitochondrial dynamics to provoke oxidative stress, DNA damage, and apoptosis; (3) Altering cortical granule distribution to hinder sperm-oocyte fusion (41, 42). Given this mechanistic evidence and our observed trend of PFOS accumulation, we propose PFOS exposure contributes to POR pathogenesis in this TCM pattern.

PFNA, an unrestricted long-chain PFAS with a 9-carbon backbone widely used in firefighting foams, metal coatings, and imaging additives, exhibits heightened toxicity despite lower human blood concentrations than PFOA/PFOS. Our data revealed marginally elevated PFNA levels in the follicular fluid of Kidney Deficiency-Liver Stagnation Pattern POR patients versus controls (1.29 vs. 1.21 nmol/L, *P* > 0.05). Mechanistically, Jiao et al. demonstrated PFNA disrupts oocyte maturation by impairing germinal vesicle breakdown and polar body extrusion through dual pathways: (1) inducing DNA damage via ROS-triggered oxidative stress; (2) causing severe spindle defects and chromosomal misalignment in MII oocytes (43). We thus propose PFNA contributes to POR pathogenesis by compromising oocyte quality through sublethal mitochondrial stress and meiotic disorganization, ultimately diminishing ART success in affected patients.

PFOA, an 8-carbon PFAS with high persistence and bioaccumulation potential, enters humans via respiratory, digestive, and dermal routes, binding plasma albumin to disrupt endocrine and reproductive functions. Our study observed a marked elevation of follicular fluid PFOA in Kidney Deficiency-Liver Stagnation Pattern POR patients versus controls (28.2 vs. 19.1 nmol/L, *P* > 0.05). Experimental evidence reveals PFOA compromises female reproduction through multisystem toxicity: suppressing ovarian steroidogenesis to impair folliculogenesis (44), inducing mitochondrial dysfunction and γ-H2AX-marked DNA damage in oocytes with consequent meiotic defects (45), and triggering granulosa cell apoptosis via disrupted gap junctional intercellular communication (46). We therefore propose that elevated PFOA contributes to POR pathogenesis in this TCM pattern through tripartite mechanisms: dysregulating ovarian hormone synthesis, compromising oocyte metabolic integrity, and impairing granulosa-oocyte crosstalk.

PAUnA, an understudied PFAS congener with recognized human toxicity, showed a non-significant upward trend in the follicular fluid of Kidney Deficiency-Liver Stagnation Pattern POR patients versus controls (1.56 vs. 1.50 nmol/L, *P* > 0.05). Given its bioaccumulative potential, we hypothesize chronic PAUnA exposure may contribute to POR pathogenesis through progressive bodily accumulation, ultimately disrupting ovarian function and impairing oocyte developmental competence.

Current evidence on PFAS exposure and fertility outcomes remains inconsistent. A Chinese prospective cohort study found no significant associations between serum/follicular fluid PFAS levels and key ART endpoints—including oocyte maturation rate, fertilization rate, high-quality embryo rate, biochemical pregnancy, clinical pregnancy, or preclinical spontaneous abortion (47). McCoy et al. conversely reported reduced blastocyst conversion rates with follicular fluid PFNA and PFDA exposure, though no links to fertilization or pregnancy outcomes were observed (48). Notably, Ma et al. demonstrated maternal plasma PFOA inversely correlated with oocyte yield and 2PN embryos, while paternal PFOA levels reduced 2PN zygote formation (49). A Singaporean case-control study further revealed 5-10% decreased fecundability per quartile increase in preconception PFDA, PFOS, PFOA, and PFHpA, with PFAS mixtures reducing clinical pregnancy and live birth likelihood by 30-40% per quartile; however, PFHxS, PFNA, and PFHpS showed no associations (18). This heterogeneity may be attributable to limited sample sizes, demographic variability across study populations, and uncontrolled confounding factors.

While prior research predominantly focused on exposure-outcome relationships without addressing therapeutic interventions, this study pioneers the evaluation of QZYST efficacy specifically for POR patients presenting with Kidney Deficiency-Liver Stagnation Pattern. POR—a pathological condition characterized by suboptimal ovarian response to gonadotropin stimulation during IVF-ET—necessitates escalated Gn dosage and prolonged stimulation to achieve adequate follicular development on hCG day. This not only imposes significant financial burdens but also heightens risks of ovarian hyperstimulation syndrome (OHSS) and luteal phase deficiency, consequently increasing early pregnancy loss rates (21).

From a TCM perspective, Professor Sun Zhengao identifies POR as a disorder rooted in ovarian dysfunction attributable to concurrent kidney and liver system pathologies. The core pathogenesis involves Kidney essence depletion, Kidney Qi deficiency, and Liver Qi stagnation as pivotal contributing factors. Drawing on decades of clinical expertise, Professor Sun empirically developed QZYST to treat POR with Kidney Deficiency-Liver Stagnation Pattern. This formula aims to tonify the Kidney and regulate Liver Qi, thereby ameliorating clinical manifestations and enhancing reproductive outcomes. The intervention duration of 31–33 days was strategically designed to bridge the transition from the preceding cycle’s luteal phase to the current stimulation cycle. This ‘pretreatment’ or ‘priming’ window is consistent with TCM principles of preparing the ovarian microenvironment to enhance oocyte competence. Physiologically, this period covers the final stages of antral follicle recruitment, providing an adequate timeframe for QZYST to exert its antioxidant and metabolic regulatory effects. QZYST comprises 13 botanical drugs, each substantiated by extensive contemporary pharmacological research.

*Rehmanniae Radix Praeparata* contains bioactive constituents including iridoid glycosides, phenylethanoid glycosides, carbohydrates, and 5-hydroxymethylfurfural (5-HMF)—a Maillard reaction product formed during processing. Crucially, enhanced follicle-stimulating hormone receptor (FSHR) expression improves granulosa cell function, whereas FSHR silencing induces apoptosis and follicular atresia (50). *Rehmanniae Radix Praeparata* may elevate 5-HMF concentrations and upregulate FSHR levels, thereby promoting granulosa cell proliferation, folliculogenesis, and ultimately ovarian reserve parameters (51). Given that dysregulated free radical metabolism impairs follicular development, ovarian reserve, and oocyte quality, 5-HMF’s free radical scavenging capacity confers significant antioxidant effects (52). Collectively, *Rehmanniae Radix Praeparata* exhibits pharmacological properties that enhance granulosa cell viability, combat oxidative stress, and mitigate cellular senescence. Additionally, its immunomodulatory and anti-inflammatory actions may benefit POR patients with autoimmune comorbidities (51, 53).

*Cornu Cervi Degelatinatum*, primarily composed of calcium carbonate, calcium phosphate, colloids, nitrogen compounds, and multiple amino acids, demonstrates significant immunomodulatory effects. Both water-processed and vinegar-processed forms reduce pro-inflammatory cytokines (TNF-α, IL-6) while elevating anti-inflammatory cytokines (IL-10) in rat models of ulcerative colitis with Yang Deficiency of Spleen-Kidney Syndrome (54). It is postulated that this dual-processed material may enhance oocyte developmental competence by modulating immune-inflammatory responses within the ovarian microenvironment.

*Cistanches Herba* contains bioactive compounds including echinacoside, verbascoside, and salidroside, which collectively confer ovarian protection by suppressing granulosa cell apoptosis through modulation of Bcl-2 family-mediated mitochondrial pathways (55). Specifically, salidroside protects dihydrotestosterone (DHT)-induced human granulosa-like tumor cells (KGN) by activating the AMPK/Nrf2 axis to attenuate oxidative stress and apoptotic cascades (56). Furthermore, phenylethanoid glycosides from *Cistanche deserticola* demonstrate hepatoprotective efficacy via triple mechanisms: stimulating hepatocyte proliferation/regeneration, while suppressing inflammation, oxidative damage, and programmed cell death (57, 58).

*Ecliptae Herba* and *Fructus Ligustri Lucidi* collectively form Erzhi Pill (EZP), a classic TCM formula with established anti-aging properties. EZP mitigates pseudo-hypoxic microenvironments and inflammatory states by downregulating HIF1α to delay aging (59). Notably, ecliptasaponin A (from *Ecliptae Herba*) and specnuezhenide (from *Fructus Ligustri Lucidi*) upregulate ESR1 to protect against cyclophosphamide-induced damage in human KGN (60). Furthermore, EZP exerts hepatoprotective effects by inhibiting PI3K/Akt/Raptor/Rictor signaling to suppress hepatocyte apoptosis (61).

*Curcumae Radix* contains key bioactive constituents including curzerene, curcuminoids, and terpenoids. Curcuminoids enhance ovarian function through anti-inflammatory, anti-apoptotic, and antioxidant properties (62), notably by boosting antioxidant enzyme activity and activating the Nrf2-Keap1 pathway to increase cellular oxidative stress resistance and survival rates (63). In D-galactose-induced premature ovarian insufficiency mice, curcumin ameliorated oxidative damage and apoptosis, elevating SOD while reducing MDA levels and downregulating SOD2/CAT mRNA expression (64). This coincided with upregulated Nrf2 and HO-1 proteins—critical mediators of ROS scavenging (64). Additionally, *Curcumae Radix* exerts hepatoprotection in CCl_4_-induced liver injury by suppressing NF-κB activation, downregulating pro-inflammatory cytokines (TNF-α, IL-1β, IL-6), and upregulating anti-inflammatory IL-10 to mitigate inflammation and fibrosis (65).

Modern pharmacological studies confirm that *Lycii Fructus* (66, 67), *Fructus Ligustri Lucidi* (68), *Fructus Mori* (69), *Rubi Fructus* (70), *Cuscutae Semen* (71), *Nelumbinis Plumula* (72), and *Rosae Laevigatae Fructus* (73) collectively exhibit antioxidant and anti-aging properties, which mitigate oocyte senescence and reduce apoptosis. In TCM, these seven botanicals—unified as “Qizi” (Seven Seeds) due to shared Kidney-tonifying and Essence-nourishing functions—all belong to seed-derived drugs. Additionally, *Rubi Fructus* (74), *Cuscutae Semen* (75, 76), *Nelumbinis Plumula* (77), and *Rosae Laevigatae Fructus* (78) demonstrate hepatoprotective effects through distinct mechanisms.

*Radix Glycyrrhizae* contains bioactive triterpenoids and flavonoids that confer potent antioxidant and immunoenhancing effects, as substantiated by pharmacological studies (79, 80).

QZYST significantly ameliorated Kidney Deficiency-Liver Stagnation Pattern manifestations in POR women, evidenced by a marked reduction in total syndrome scores post-intervention (median [IQR]: 18 (15, 22) vs. 21 (18, 24); *P* < 0.001). Compared with the POR-NON group, QZYST-treated subjects demonstrated substantially lower syndrome scores (18 (15, 22) vs. 21 (19, 23); *P* < 0.05), confirming its therapeutic efficacy for this TCM pattern. QZYST treatment significantly improved key manifestations of Kidney Deficiency-Liver Stagnation Pattern (all *P* < 0.05), including: premenstrual irritability or chronic depression, soreness in lumbar and knees, dizziness and tinnitus, premenstrual breast distension, loss of libido and dark tongue body. Although symptomatic relief for scanty menstruation, darkish or dull menstrual blood, hot flashes with sweating, mental fatigue and weakness, insomnia with dreamfulness, hypochondriac distension/pain, clear profuse urine and deep thready or wiry pulse did not reach statistical significance (*P* > 0.05), amelioration trends were observed versus baseline. In the present study, the significant improvements in the number of oocytes retrieved and 2PN zygotes, coupled with a reduced rate of cycles with no transferable embryos, underscore the clinical utility of QZYST for POR patients. Although the clinical pregnancy and cumulative pregnancy rates showed only a modest numerical increase without statistical significance, this is likely attributable to the limited sample size, as binary pregnancy outcomes require larger cohorts to achieve sufficient statistical power. For POR patients—a population characterized by a low prognosis—the ability to obtain more oocytes and embryos is the fundamental prerequisite for any successful pregnancy (81). Our findings suggest that QZYST primarily acts by optimizing the follicular microenvironment and reducing the toxic impact of PFAS, thereby expanding the pool of available embryos for transfer.

Additionally, in the follicular fluid of patients with Kidney Deficiency-Liver Stagnation Pattern POR who received QZYST intervention (POR-TCM group), the level of PFBA was significantly lower than that in POR patients without Chinese herbal medicine intervention (POR-NON group), and the difference was statistically significant (P < 0.05). It is important to acknowledge that the observed association between reduced follicular PFBA levels and improved clinical outcomes in the POR-TCM group remains correlative. In the complex biological system of the ovary, QZYST likely exerts a synergistic effect by both enhancing the metabolic clearance of EEDs like PFBA and directly attenuating oxidative stress within granulosa cells. While our data suggest that the reduction of PFAS burden is a key metabolic feature of successful TCM intervention, further functional experiments—such as utilizing PFAS-exposed cell models or animal studies—are required to definitively establish a causal link and map the downstream molecular signaling pathways. However, although the levels of five other perfluoroalkyl substances—PDA, PFOS, PFNA, PFOA, and PAUnA—also showed a downward trend in the POR-TCM group, the differences were not statistically significant (P > 0.05). Therefore, we speculate that the improvement in TCM syndrome manifestations and clinical outcomes after QZYST intervention may be associated with changes in the levels of certain PFAS compounds in follicular fluid.

## Limitation

This study has several limitations. First, the non-randomized and sequential recruitment design might introduce time-related bias, although our clinical protocols and laboratory standards remained strictly consistent throughout the study period. Second, while the sample size was small due to high testing costs, our post-hoc power analysis confirmed that the data is robust for the main PFAS marker (power=0.89 for PFBA), even though the power for clinical outcomes was more moderate (0.53). Third, we did not use a placebo or measure baseline PFAS levels before treatment, which makes it difficult to strictly prove a cause-and-effect relationship or rule out psychological effects on subjective symptom scores. Fourth, we only tested follicular fluid without paired blood or urine samples, so we cannot determine if the medicine cleared PFAS from the whole body or specifically protected the ovaries. Finally, due to time and funding limits, we did not perform cell experiments to explore the exact biological mechanisms, nor did we track long-term live birth rates. Future large-scale, placebo-controlled studies with multi-matrix sampling and functional experiments are needed to confirm these preliminary findings.

## Conclusions

This study suggests a potential association between elevated follicular PFAS levels and the pathogenesis of POR. QZYST intervention was associated with a reduction in TCM syndrome scores and a higher number of oocytes retrieved and 2PN zygotes, along with lower levels of PFAS concentrations. These findings support QZYST as an effective and safe complementary therapeutic option for managing this specific POR phenotype. Future research should explore long-term outcomes and potential pharmacokinetic interactions of QZYST within broader POR treatment paradigms to comprehensively define its clinical utility.

## Data Availability

The raw data supporting the conclusions of this article will be made available by the authors, without undue reservation.
