# Correction: Association between PFAS compounds in follicular fluid and POR and the intervention effect of Qizi Yusi Tang: a study based on targeted PFAS quantification analysis

**DOI:** 10.3389/fendo.2026.1825216

**Published:** 2026-03-18

**Authors:** Hengbing Li, Jiacheng Li, Wenjing Jiang, Xin Hu, Zizhen Guo, Jingyan Song, Zhengao Sun

**Affiliations:** 1 The First Clinical Medical College, Shandong University of Traditional Chinese Medicine, Jinan, China; 2 Department of Thoracic Surgery, Qilu Hospital of Shandong University, Jinan, China; 3 School of Medicine, Tongji University, Shanghai, China; 4 Reproductive Center of Integrated Medicine, Affiliated Hospital of Shandong University of Traditional Chinese Medicine, Jinan, China

**Keywords:** non-randomized prospective sequential comparative design, poor ovarian response, Qizi Yusi Tang, targeted per- and polyfluoroalkyl substances analysis, traditional Chinese medicine

In the published article, the affiliation number assignment for multiple authors was erroneous.

The original incorrect author list and affiliation mapping were displayed as:

“Hengbing Li^1†^, Jiacheng Li^2†^, Wenjing Jiang^2^, Xin Hu^3^, Zizhen Guo^1^, Jingyan Song^4*^ and Zhengao Sun^4*^

^1^The First Clinical Medical College, Shandong University of Traditional Chinese Medicine, Jinan, China

^2^Department of Thoracic Surgery, Qilu Hospital of Shandong University, Jinan, China

^3^School of Medicine, Tongji University, Shanghai, China

^4^Reproductive Center of Integrated Medicine, Affiliated Hospital of Shandong University of Traditional Chinese Medicine, Jinan, China”

The correct author list and affiliation mapping are as follows:

“Hengbing Li^1†^, Jiacheng Li^2†^, Wenjing Jiang^3^, Xin Hu^1^, Zizhen Guo^4^, Jingyan Song^4*^ and Zhengao Sun^4*^

^1^The First Clinical Medical College, Shandong University of Traditional Chinese Medicine, Jinan, China

^2^Department of Thoracic Surgery, Qilu Hospital of Shandong University, Jinan, China

^3^School of Medicine, Tongji University, Shanghai, China

^4^Reproductive Center of Integrated Medicine, Affiliated Hospital of Shandong University of Traditional Chinese Medicine, Jinan, China”

The original version of this article has been updated.

